# Comparison of Marginal and Internal Adaptation in Endocrowns Milled from Translucent Zirconia and Zirconium Lithium Silicate

**DOI:** 10.1155/2021/1544067

**Published:** 2021-12-07

**Authors:** Amirhesam Amini, Somayeh Zeighami, Safoura Ghodsi

**Affiliations:** ^1^Dental Research Center, Dentistry Research Institute, Tehran University of Medical Sciences, Tehran, Iran; ^2^Department of Prosthodontics, Faculty of Dentistry, Tehran University of Medical Sciences, Tehran, Iran

## Abstract

**Purpose:**

This study aimed to compare marginal and internal adaptation in endocrowns made from translucent zirconia and zirconium lithium silicate using CAD-CAM technology.

**Materials and Methods:**

Twenty-eight freshly extracted upper molars were mounted in acrylic resin and underwent root canal therapy and endocrown preparation up to 2 mm above the cementoenamel junction. Endocrowns were CAD-CAM milled from zirconium lithium silicate (ZLS) and translucent zirconia (Zr). Internal and marginal adaptation was assessed by the replica technique before cementation. Marginal adaptation was evaluated by a stereomicroscope (×32) before and after cementation and also after thermomechanical aging.

**Results:**

The ZLS group showed significantly higher internal adaptation compared to the Zr group (*P* = 0.028), while the marginal adaptation differences, at different times with different methods, were not statistically significant (*P* > 0.05). Axiomarginal angle had the highest and axiopulpal angle showed the lowest adaptation in both groups. The cementation process and thermomechanical aging increased the marginal gap in both groups significantly (*P* < 0.001). The marginal gap assessed by the replica technique before cementation was 7.11 *µ*m higher than direct view under a stereomicroscope with intraclass correlation coefficient of 0.797.

**Conclusion:**

Zirconia seems to be an acceptable material for endocrown with comparable internal and marginal adaptation to ZLS. Cementation and thermomechanical aging had significantly negative effects on marginal gap. The marginal gap assessed by the replica technique was higher than direct view under the stereomicroscope technique.

## 1. Introduction

Functional requirements and the extent of coronal destruction are the two most important determinants in selecting the most efficient way of restoring endodontically treated teeth [1]. Since the main purpose of dowel post application in the most prevalent method of restoring these teeth, post core and crown, is providing retention for the crown superstructure, it will be remarkable to provide sufficient retention without the need of tooth preparation, post application, and their related consequences [2]. Endocrown, introduced in 1995 by Pissis, was a breakthrough in the constant challenge of rehabilitating endodontically treated teeth [3]. This adhesively retained monoblock ceramic restoration plays the role of post, core, and crown at the same time [4, 5]. Being more conservative, having superior aesthetics, and requiring less clinical and laboratory steps and time compared to conventional crown are some of the advantages of this technique [6–9]. In the main concept, endocrown benefits from both macro and micromechanical retention while primarily relying on microretention [10–12]. Therefore, to this date, the majority of the materials used in this type of restorations had the potential of being adhesively bonded to the tooth structure (e.g., glass containing ceramics) [13]. Zirconium lithium silicate, which is manufactured by adding zirconium dioxide to lithium silicate glass ceramic, is one of the newly developed glass ceramics with excellent optical and mechanical properties [14,15].

Zirconia, a nonglass containing polycrystalline ceramic, has drawn lots of attention to itself due to the superb mechanical properties and development of digital dentistry [16–19]. Special characteristics of zirconia and manufacturing translucent types have made it one of the prime choices for lots of restorations [16]. Due to the absence of glassy phase in the structure and the fact that it is not etchable, application of zirconia in adhesively retained restorations like endocrown is not common. In fact, the efficacy of zirconia in restorations like endocrowns has been evaluated only in a few short-term clinical studies [20, 21]. However, translucent zirconia has been recently suggested as a potential endocrown material to be used in high-stress situations [21–23]. Although the dimensional changes of zirconia during the sintering process (20–30% [24]) is predicted and compensated in the designing stage, it may affect the accuracy and the final adaptation of restorations. Therefore, evaluating the adaptation of zirconia endocrown is among the necessary assessments before accepting it as a standard ceramic in endocrowns.

Acceptable internal/marginal adaptation plays a major role in predictable long-term clinical service of fixed restorations [25–31]. Adaptation can be evaluated by measuring internal and marginal gaps. Internal gap, according to Holmes et al. [32], is defined as the perpendicular distance from the internal surface of restoration to the axial wall of preparation, whereas the same measurement at the margin is called marginal gap.

The accuracy of a CAD-CAM fabricated restoration is determined by a variety of factors such as machinability, postmilling dimensional changes, and determined cement space. This index can be investigated by different methods, from simple ones like using an explorer to advanced techniques like micro-CT [33–40]. There are studies on comparing the adaptation of glass ceramics and zirconia in crowns with controversial results, while there are no such studies on endocrowns. Considering differences between crown and endocrown regarding preparation design, area covered by the restoration, finish line width, and shape of the restoration, the primary objective of this study was to compare the internal and marginal adaptation of CAD-CAM fabricated endocrowns made of zirconium lithium silicate (ZLS) and translucent zirconia (Zr) before and after cementation and thermomechanical aging. The secondary objectives were to assess the effect of cementation, aging, and measurement technique on marginal adaptation. The null hypotheses were that there is no significant difference in internal or marginal adaptation between Zr and ZLS, and cementation process, thermomechanical aging, and the measurement method have no significant effects on marginal gap.

## 2. Materials and Methods

The study protocol was registered and approved in the institutional ethics committee (IR.TUMS.DENTISTRY.REC.1399.050). Twenty-eight extracted maxillary molars, extracted for orthodontic and periodontal reasons, were collected in a period no more than two months prior to the study and stored in Hank's balanced saline solution which was replaced every week. Using the Vernier caliper (INSIZE, Suzhou, China), the teeth were selected based on dimensional range of 10–12 mm in buccolingual and 9–11 mm in mesiodistal directions. The other inclusion criteria were absence of caries, restorations, or cracks. Plaque and calculus were removed by a brush and ultrasonic scaler to simulate the oral environment conditions.

**Figure 1:**
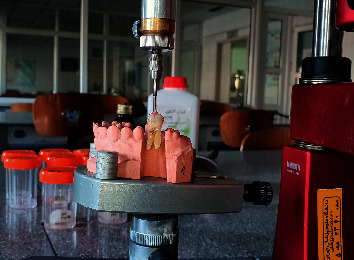
The process of mounting tooth specimen using a surveyor (the tooth is not in its final position).

After making impression of a typodont, upper and lower casts were poured by type 4 dental stone (Asia Chemi Teb Pharmaceutical Co., Tehran, Iran). A trapezoidal space was prepared in the first molar area in each side of the maxillary cast to be used as a mold for the specimens. The tooth specimens were mounted vertically by a surveyor in self-cure acrylic resin in occlusion with the mandibular cast (Figure 1) and scanned by a laboratory scanner (inEos X5; Dentsply Sirona, York, PA) to acquire biogeneric copies to replicate tooth's preoperative shape.

**Figure 2:**
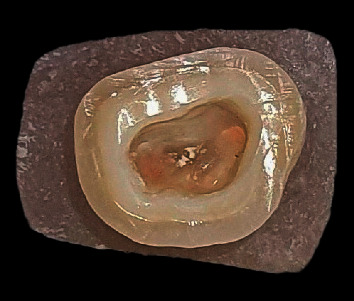
Prepared molar tooth specimen.

Standard root canal therapy was performed by the lateral condensation method and the canals were filled. Afterwards, the teeth were prepared for endocrown up to 2 mm above the cementoenamel junction to simulate extensive tissue loss (as the worst-case scenario) with 90 degrees butt margin and 12 degrees total wall divergence using wheel diamond burs (JOTA AG, Rüthi, Switzerland) and cylindrical diamond burs (JOTA AG, Rüthi, Switzerland) and a handpiece mounted on a surveyor to control the angulation and contour of the preparation. The remaining walls under 2 mm width were reduced. A periodontal probe confirmed the depth of 3–5 mm in the pulp chamber; otherwise, the tooth was excluded. The margins were polished using composite polishing diamond burs and composite polishing rubber disks (JOTA AG, Rüthi, Switzerland). Finally, the pulp chamber was cleaned with 95% ethanol (Figure 2).

The prepared specimens (*n* = 28) were randomly divided into two groups by stratified random allocation and scanned again. The endocrowns were designed using biogeneric copy protocol (inLab CAD SW; Dentsply Sirona, York, PA). Cement space was set at 0 for the margins and 50 *µ*m for other areas. The restorations were milled from ZLS (Celtra Duo; Dentsply Sirona, York, PA) and high-translucent Zr (DD Bio ZX^2^; Dental Direkt, Spenge, Germany) by a milling machine (inLab MC X5; Dentsply Sirona, York, PA) (*n* = 14 in each group). Both materials had A2 shade according to the VITA system. A summarized description of the materials used in this study is presented in Table 1. Milling burs were replaced on the machine warning. ZLS restorations were recrystallized and glazed (at 820°C), and Zr restorations were sintered (at 1450°C) and glazed according to the manufacturer's instructions.

**Figure 3:**
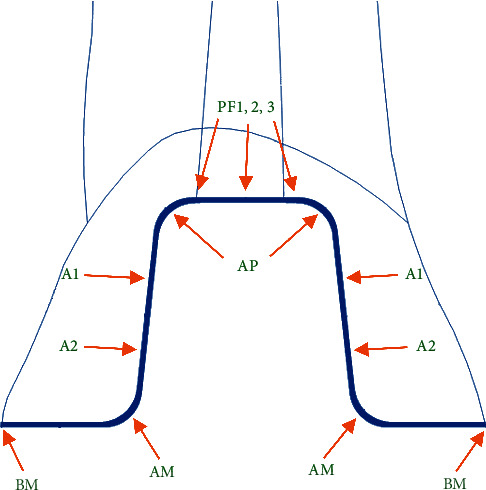
Schematic drawing showing points assessed in the replica technique (PF, pulpal floor; AP, axiopulpal angle; A, Axial wall; AM, axiomarginal angle; BM, butt margin).

The seat of each restoration was assessed (Fit checker; GC, Tokyo, Japan). Except one of the restorations in the ZLS group, other endocrowns did not need any adjustments. Passive fit was confirmed by two impartial observers. For measuring the internal and marginal gap by the replica technique, the intaglio surface of each restoration was covered with a low viscosity silicone impression material (Panasil initial contact; Kettenbach, Eschenburg, Germany), and the restoration was seated on its corresponding tooth. To simulate the cementation process, the tooth-restoration complex was held under a 5 kg weight until the impression material was set. Afterwards, the endocrown was separated while the impression material was left on the tooth. A high viscosity polyvinyl siloxane (3M Express VPS; 3M, Saint Paul, MN) was injected on the set light material to support the replica specimen. The replica was cut buccolingually and mesiodistally and assessed by the stereomicroscope (SZX16; Olympus, Tokyo, Japan) under ×32 magnification using Microbin software (Microteb, Tehran, Iran) at the points shown in Figure 3.

**Figure 4:**
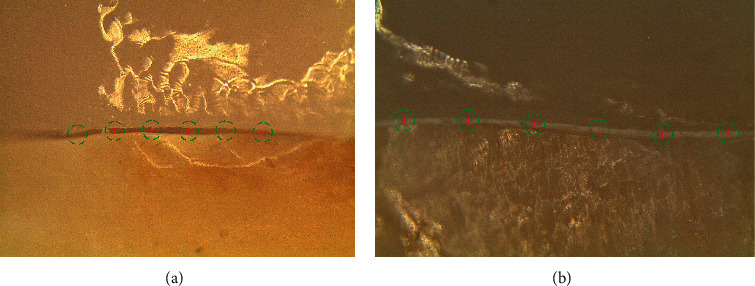
Stereomicroscope photographs of two specimens. (a) Before cementation. (b) After cementation. Red lines show gap/cement layer thickness measurements.

Marginal gap was also assessed directly under the stereomicroscope while a clamp held the restoration and the tooth together (Figure 4(a)). Each specimen was assessed at 12 marked points (3 points on each surface). The assessment points were marked by #11 surgical blade on the CEJ of the teeth to ensure repeating the subsequent measurements at the same points.

The ZLS specimens were prepared using hydrofluoric acid (Cera-Etch; Morvabon, Tehran, Iran) and silane (Master-Dent Porcelain Primer; Dentonics, Charlotte, NC), while sandblasting by 50 *µ*m Al_2_O_3_ was used for Zr. Equal amounts of ED Primer II A and B liquids (Kuraray, Tokyo, Japan) were mixed and applied on bonding surfaces of the tooth, left for 30 s, and then dried gently by air flow. Afterwards, equal amounts of Panavia F2.0 A and B pastes (Kuraray, Tokyo, Japan) were mixed for 20 s, applied on bonding surfaces of each restoration, and the endocrown was seated on the corresponding tooth. Under a 5 kg weight, each specimen was light-cured (LED.D; Guilin Woodpecker Medical Instrument Co., Guangxi, China) for 5 s, and the excess cement was removed using an explorer. Finally, each tooth surface was light-cured for 20 s. Cemented specimens were kept in an incubator at 37°C for 24 h. Marginal gap was reassessed under the stereomicroscope after cementation (Figure 4(b)).

The specimens underwent thermomechanical aging, 5000 thermal cycles (C-300; Vafaei Industrial Factory, Qom, Iran) at 5°C and 55°C with 25°s dwell time, which simulated 6 months of clinical usage and 500,000 loading cycles (Chewing Simulator CS-4; SD Mechatronik, Feldkirchen-Westerham, Germany) with 50 N force and 1.64 Hz frequency by a metal sphere with 4 mm diameter in 100% humidity environment, which simulated 24 months of clinical usage. Marginal gap was assessed by the direct technique for the third time.

The data were analyzed using SPSS 22.0 (IBM, Armonk, NY). The paired samples *t*-test was performed to assess the effect of the measurement method on the marginal gap before cementation (replica vs. the direct technique). The effect of material (ZLS vs. Zr) on internal and marginal adaptation was assessed by the independent samples *t*-test, which was also employed to compare gaps in different areas within each group. The effect of cementation and thermomechanical aging on marginal gap was evaluated using repeated-measures ANOVA.

## 3. Results

Descriptive results for internal gaps measured by replica technique are given in Table 2.

The independent samples *t*-test showed a significant difference between the two groups (*P* = .028), where ZLS had better overall internal adaptation (lower internal gap). There were also significant differences between different areas in each group (*P* < .05) except for the difference between axiopulpal angle and pulpal floor in the ZLS group (*P* = .478). In both groups, axiopulpal line angle had the highest internal gap followed by pulpal floor and axial wall, while axiomarginal line angle had the least. There were statistically significant differences between ZLS and Zr regarding the gap measured at pulpal floor and axiopulpal angle (*P* < .05). Internal adaptation at axial wall (*P* = .125) and axiomarginal angle (*P* = .580) indicated no significant difference between groups.

The mean marginal gaps are presented in Table 3.

Table 4 provides the marginal gap at different surfaces. The independent samples *t*-test showed no statistically significant difference regarding measurement methods, material type, and different surfaces between evaluated groups (*P* < .05).

**Figure 5:**
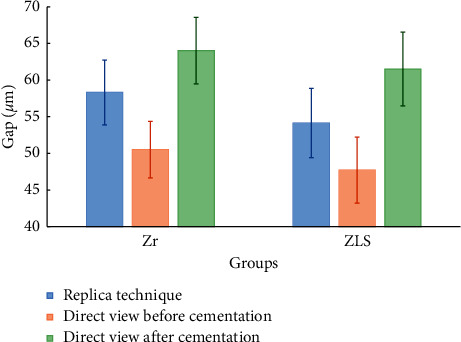
Mean and 95% confidence interval of replica and direct view marginal gap measurements before and after cementation.

Repeated measures ANOVA indicated that cementation and thermomechanical aging had significant effects on increasing marginal gap in both groups (*P* < .001).  Figure 5 shows the comparison between replica and direct view technique in measuring marginal gap before and after cementation.

*T*-test analysis showed a statistically significant difference in this comparison (*P* < .001) in which the replica technique measured marginal gaps 7.11 *µ*m (on average) greater than the direct method with intraclass correlation coefficient of 0.797. Furthermore, the marginal gap measured by the replica technique before cementation was averagely 6.53 *µ*m less than the direct method after cementation.

## 4. Discussion

Internal and marginal adaptation, as the accuracy indices of restorative process, have significant clinical implications. Poor marginal adaptation (higher marginal gap) could result in the exposure of cement to oral fluids, leakage, plaque accumulation, secondary caries, periodontal inflammation, and eventually, complete failure of prosthodontic treatment [27–29]. Inadequate internal adaptation (higher internal gap), on the other hand, will lead to increased stress at the interface between tooth and restoration and possibly fracture of the restoration [30]. CAD-CAM fabricated restorations generally show higher (better) adaptation compared to conventional workflow [41]; however, their accuracy might be influenced by the type of scanner and milling machine, cement space, preparation design, microstructure and machinability of the material, and dimensional changes after milling [33–35].

The present study focused on evaluating the accuracy of translucent zirconia as a potential material in fabricating partial-coverage restorations and comparing it with zirconium lithium silicate as a routine glass ceramic for endocrown fabrication. To simulate the dynamic oral environment, thermomechanical aging was applied. Furthermore, the adaptation of restorations was assessed by two techniques (replica and direct view under the stereomicroscope) to evaluate the possible effect of the measurement method on final results.

The null hypotheses were partially rejected since there was a significant difference in internal gap results between materials and surfaces and between the marginal gap measurements obtained by different techniques.

ZLS had significantly lower internal gap which was significantly less in axiomarginal angles and axial walls compared to pulpal floor and angles. This result was consistent with the results presented by Shin et al. [36], Hasanzade et al. [26], and Zimmermann et al. [42]. This significant difference could be attributed to the surface anatomy, where pulpal floor has more uneven surface compared to axial wall, and the distance from the scanner head, where deep pulpal cavity will lead to higher internal gaps. In other words, limited optical depth of the scanner and receiving blurred image of pulpal floor and axiopulpal line angles alongside their unsmooth surface anatomy are the reasons for less accuracy (higher gaps) in these areas [26, 42, 43]. More surface details (canal orifices, remaining obturation materials, and surface irregularities) in pulpal floor and axiopulpal line angle could also lead to overmilling of the restoration by the milling machine, which results in higher internal gaps. The significant difference between Zr and ZLS groups may be attributed to the different physical properties of the materials such as hardness and their different firing shrinkage rates. The sintering shrinkage of Zr has been reported between 20% and 30%, while ZLS has 0.5% firing shrinkage [24,37]. Also, difference in hardness can affect the machinability in the milling system [14, 15, 18, 19]. El Ghoul et al. indicated that there is an inverse relation between hardness and machinability of a material [43]. However, brittleness index seems to be a more precise indicator which is obtained by dividing hardness to fracture toughness [38, 39]. Since there is no study on comparing the hardness and the brittleness indices of Celtra Duo and DD Bio ZX^2^ brands, the difference in internal gaps could not be attributed to these characteristics with certainty.

Marginal gap measurements showed no significant differences between Zr and ZLS; however, ZLS had slightly higher (better) marginal adaptation. Zirconia restorations generally show less marginal gap compared to internal gap [16]. This could be attributed to the shape or size of milling burs and less anatomical complexity at the margins, especially butt margin which was used in the present study [16].

Cementation had a significant effect on marginal gap in both groups, which is similar to the results obtained by Taha et al. [44]. Cement film thickness, although in acceptable clinical range (25 *µ*m) [45], might justify this significant change. Thermomechanical aging had a similar effect on marginal gap and significantly increased gap in both groups. It has been suggested that cyclic loading and thermal cycling exert thermomechanical stresses on the cement layer where its deterioration leads to increased marginal gap [44]. This result is against Kassem et al. who indicated thermomechanical aging reduced marginal gap [46]. This contradiction could be justified by the characteristics of different materials used. Higher resiliency of hybrid ceramics used in Kassem et al.' study could affect the stress transfer and the materials dimensional change under loading pressure.

Comparing marginal gaps before cementation assessed by different techniques showed that the replica technique measured marginal gaps 7.11 *µ*m more than direct view under the stereomicroscope on average, and the difference was significant. This has been explained by assuming that replica material has a similar effect on marginal gap as a cement does [40]. Also, comparing replica results before cementation with the marginal gap measurements obtained after cementation showed 6.53 *µ*m difference where replica results were less. This comparison emphasizes the importance of considering the effects of the measuring method in analyzing the results obtained in research studies. It is noteworthy to mention that all the marginal gaps measured in this study using different methods at different times were within clinically acceptable range (120 *µ*m [31]).

The present study showed that Zr could be an acceptable endocrown material with regard to final accuracy. Translucent zirconia has attracted a lot of attention recently and could be an outstanding material option in all types of fixed restorations including partial-coverage ones provided that its characteristics (retention, adaptation, fracture resistance, and failure mode) get confirmed in scientific research studies.

The present study used a poly vinyl siloxane impression material with light viscosity in the replica technique. Using lower viscosities (x-light) might lead to different results. Also, more thermomechanical aging cycles which simulates long-term usage in oral environment might bring about some changes to the results. Therefore, the effect of different consistencies of replica materials and cements on marginal gap with more aging cycles obtained by different methods calls for further studies.

## 5. Conclusions

Within the limitations of this in vitro study, following conclusions were obtained:

1. In terms of accuracy and adaptation, zirconia seems to be a suitable material for fabricating endocrowns
2. Both cementation and thermomechanical aging had significant negative increasing effects on marginal gaps in both groups
3. Before cementation, the replica technique showed 7.11 *µ*m higher measurements than direct view under the stereomicroscope technique, which indicates the method of marginal gap assessment could affect the final results of a study

## Figures and Tables

**Table 1:** The physical properties of ceramic materials used in the study.

Group	Ceramic type	Flexural strength	Fracture toughness (SEVNB)	E-modulus	Vickers hardness	Brittleness index
ZLS (Celtra Duo)	Zirconium lithium silicate ceramic	Mill and polish: 210 MPa^*∗*^	2 MPa m^0.5^^*∗*^	Approx. 70^*∗*^	6.86 GPa^*∗*^	2.84 *µ*m^−1/2†^ [15]
		Mill and fire: 370 MPa^*∗*^				
Zr (DD Bio ZX^2^)	High-translucent zirconium oxide ceramic	1100–1250 MPa^*∗*^	>10 MPa m^0.5^^*∗*^	>200^*∗*^	5.41–15.47 GPa^†^ [18, 19]	1.52–3.15 *μ*m^−1/2†^ [18]

^*∗*^According to the manufacturer's claim. ^†^According to the available data on the same material type, but different brand, due to the lack of data on the particular brand used in the present study.

**Table 2:** Internal gap results.

	Area	Group	Mean	SD	Min-max	*P* value
Internal gap (*µ*m, *n* = 14)	Pulpal	Zr	99.75	±17.99	81.01–145.83	0.047
		ZLS	82.65	±24.79	58.98–149.38	
	Axiopulpal	Zr	113.81	±17.18	87.63–148.72	0.002
		ZLS	85.62	±25.27	54.13–130.88	
	Axial	Zr	78.13	±14.67	56.71–100.21	0.125
		ZLS	69.09	±15.52	47.24–98.56	
	Axiomarginal	Zr	56.42	±14.00	40.51–81.01	0.580
		ZLS	53.35	±14.95	34.37–86.12	
	Overall internal gap	Zr	87.03	±14.39	66.46–117.36	0.028
		ZLS	72.68	±18.05	51.22–112.66	

Zr, zirconia; ZLS, zirconium lithium silicate; SD, standard deviation; Min, minimum; Max, maximum.

**Table 3:** Mean marginal gaps and standard deviations before cementation (replica and direct view technique), after cementation, and after thermomechanical aging.

	Marginal gap (*µ*m, *n* = 14)							
	Before cementation replica technique		Before cementation direct view		After cementation direct view		After aging direct view	
Group	Mean	SD	Mean	SD	Mean	SD	Mean	SD
Zr	58.33	±8.43	50.52	±7.35	64.02	±8.66	78.52	±10.25
ZLS	54.14	±9.02	47.71	±8.62	61.51	±9.60	80.62	±11.72
*P* value in comparison to precementation direct view measurement					<0.001		<0.001	

Zr, zirconia; ZLS, zirconium lithium silicate; SD, standard deviation.

**Table 4:** Mean marginal gaps and standard deviations of buccal-lingual and mesial-distal surfaces (separately) at different measurement stages.

Time and method	Area	Group	Mean	SD	Min-max
Before cementation replica	Buccal and lingual	Zr	54.88	±10.35	40.51–73.06
		ZLS	50.51	±9.16	40.51–65.32
	Mesial and distal	Zr	61.77	±7.68	48.61–72.91
		ZLS	57.76	±10.59	40.51–79.79

Before cementation direct view	Buccal and lingual	Zr	50.35	±11.50	32.41–72.91
		ZLS	46.55	±9.47	33.40–64.81
	Mesial and distal	Zr	50.69	±5.60	40.51–57.29
		ZLS	48.88	±9.60	32.41–69.12

After cementation direct view	Buccal and lingual	Zr	63.70	±11.62	47.31–87.76
		ZLS	59.63	±9.21	45.47–77.40
	Mesial and distal	Zr	64.35	±9.57	49.43–89.12
		ZLS	63.39	±11.92	41.79–84.56

After aging direct view	Buccal and lingual	Zr	78.78	±11.06	62.87–101.13
		ZLS	78.74	±10.64	64.45–97.22
	Mesial and distal	Zr	78.26	±10.73	64.91–103.54
		ZLS	82.49	±14.11	57.89–113.42

Zr, zirconia; ZLS, zirconium lithium silicate; SD, standard deviation; Min, minimum; Max, maximum.

## Data Availability

The data used to support the findings of this study are available upon request from the author (amirhesamamini430@gmail.com).
